# Lipid-Based Particles: Versatile Delivery Systems for Mucosal Vaccination against Infection

**DOI:** 10.3389/fimmu.2018.00431

**Published:** 2018-03-07

**Authors:** Blaise Corthésy, Gilles Bioley

**Affiliations:** ^1^R&D Laboratory, Division of Immunology and Allergy, Centre des Laboratoires d’Epalinges, Centre Hospitalier Universitaire Vaudois (CHUV), Lausanne, Switzerland

**Keywords:** mucosal, vaccination, lipidic particles, delivery system, infections

## Abstract

Vaccination is the process of administering immunogenic formulations in order to induce or harness antigen (Ag)-specific antibody and T cell responses in order to protect against infections. Important successes have been obtained in protecting individuals against many deleterious pathological situations after parenteral vaccination. However, one of the major limitations of the current vaccination strategies is the administration route that may not be optimal for the induction of immunity at the site of pathogen entry, i.e., mucosal surfaces. It is now well documented that immune responses along the genital, respiratory, or gastrointestinal tracts have to be elicited locally to ensure efficient trafficking of effector and memory B and T cells to mucosal tissues. Moreover, needle-free mucosal delivery of vaccines is advantageous in terms of safety, compliance, and ease of administration. However, the quest for mucosal vaccines is challenging due to (1) the fact that Ag sampling has to be performed across the epithelium through a relatively limited number of portals of entry; (2) the deleterious acidic and proteolytic environment of the mucosae that affect the stability, integrity, and retention time of the applied Ags; and (3) the tolerogenic environment of mucosae, which requires the addition of adjuvants to elicit efficient effector immune responses. Until now, only few mucosally applicable vaccine formulations have been developed and successfully tested. In animal models and clinical trials, the use of lipidic structures such as liposomes, virosomes, immune stimulating complexes, gas-filled microbubbles and emulsions has proven efficient for the mucosal delivery of associated Ags and the induction of local and systemic immune reponses. Such particles are suitable for mucosal delivery because they protect the associated payload from degradation and deliver concentrated amounts of Ags *via* specialized sampling cells (microfold cells) within the mucosal epithelium to underlying antigen-presenting cells. The review aims at summarizing recent development in the field of mucosal vaccination using lipid-based particles. The modularity ensured by tailoring the lipidic design and content of particles, and their known safety as already established in humans, make the continuing appraisal of these vaccine candidates a promising development in the field of targeted mucosal vaccination.

## Introduction

Vaccination is considered as one of the most successful medical actions and has greatly contributed to the improvement of world health. Indeed, it has strikingly reduced the prevalence of many infectious diseases, and thus helps nowadays to save millions of lives each year (1, 2). Vaccine administration aims at inducing and harnessing protective effector and memory immunity, comprising neutralizing antibodies (Abs) together with cytotoxic and helper T cells (3) able to control subsequent challenge by the target pathogen. Live-attenuated or killed whole-pathogens have originally been administered for vaccination purposes, but due to safety concerns, including important reactogenicity and risks of reversion, the use of subunit vaccines is preferred. The latter are composed of recombinant or purified pathogen-derived antigenic entities, mostly depleted of innate immune stimulus, that require the co-administration of adjuvants and/or the use of delivery vehicles to achieve sufficient immunogenicity. Over the last decades, important pieces of work in the field of vaccine technology have allowed to rationally design and develop formulations that ensure efficient induction of immune responses (4). Synthetic micro-/nanoparticles, liposomes, immune stimulating complexes (ISCOMs), virosomes, virus-like particles, as well as emulsions, all offer several interesting attributes for vaccine delivery and have already proven efficient in parenteral (intramuscular or subcutaneous) vaccinations by inducing protection against infectious agents (5). These formulations have been designed to mimic biophysical and biochemical features of pathogens, thus ensuring efficient display and delivery of concentrated amounts of antigens (Ags) and adjuvants to innate and adaptive immune cells. Interestingly, this leads to reducing the number of injections required to elicit potent cellular and humoral immune responses with minimal cytotoxicity.

Despite important success in protecting individuals against many deleterious pathological situations, it remains that most of the licensed subunit vaccines are administered parenterally. However, except in previously infected individuals, such a route of administration only induces limited protective effect at the level of mucosal surfaces, the sites where the vast majority of pathogenic agents gain access to the host body (6). In addition to mechanical (epithelium covered with mucus) and chemical (anti-microbial peptides) barriers found at mucosae, adaptive humoral and cellular immunity is of prime importance to efficiently protect against pathogenic insults (7). Thus, to reinforce the efficiency of vaccination, the delivery of vaccine formulations directly to the mucosa represents an asset. It is now well accepted that immune responses have to be elicited locally to ensure efficient imprinting of effector and memory B and T cell homing to mucosal tissues where they will limit entry, colonization, and spreading of pathogens (8–11). Until now, the few licensed mucosal vaccines consist in administration of live-attenuated or killed whole-pathogens that raise similar safety concerns as for parenteral injection, while no subunit vaccines have been approved for human use. This is mainly due to technical difficulties inherent to the administration route and the physiology of the tissues where the vaccine formulations are applied. Identifying the most adequate vaccine formulation deliverable mucosally remains challenging due to (1) the fact that, in contrast to parenteral vaccination where injected Ags and adjuvants are directly in contact with antigen-presenting cells (APCs), Ag sampling has to be performed across the mucus and the epithelium first; (2) the deleterious acidic, proteolytic, and dynamic environment of the mucosal surfaces which impact the stability, integrity, and retention time of the applied Ags; and (3) the tolerogenic nature of the mucosa, which impairs induction of effector immunity to antigenic entities lacking sufficient immunostimulatory signals (12). Such hurdles may, however, be partially overcome thanks to recent progress made in the understanding of mucosal immunity and in the field of vaccine technology (6).

Apart from immunological and physiological aspects, one important point to be considered for vaccination is the compliance of the patients (13). For pediatric vaccination, administration has to be minimally invasive and easy to perform. The ability of vaccinating a large number of people in countries where endemic infections are present, but where access to medical infrastructures is limited, is of great importance as well. In this context, parenteral vaccination is not the most appropriate strategy as injections are invasive, painful and require trained/skilled medical staff for administration. Moreover, it poses problems related to the risks associated with infection at the site of injection, needle-stick injury, spreading of transmissible diseases, and disposal of used materials. Thus, there is an increasing demand for needle-free vaccination. As an example, mucosal vaccines display several advantages, such as ease of administration and self-delivery allowing mass vaccination, absence of needle-associated risks, and in some cases lower costs and simplified production due to absence of administration devices.

Until now, only few mucosally applicable subunit vaccine formulations have been developed and successfully tested (14), mainly because of the limited number of safe and efficient delivery systems and adjuvants available, coupled to the sometimes important amounts of Ag to be administered. This review will focus on lipid-based micro-/nanoparticles that possess several of the desired characteristics of an interesting Ag-delivery system for vaccination as they are biocompatible, can overcome physiological barriers at mucosae, promote Ag crossing of the epithelium and uptake by APCs, protect the associated payload, are adequate for incorporating adjuvants and may display mucoadhesive properties. In order to achieve the induction of protective anti-pathogen humoral and cellular responses at the relevant mucosal surfaces, the choice of the most potent administration route has to be carefully considered by taking into account the physiological and immunological features of the different target tissues (15). These aspects and the strategies to specifically target vaccines to the portals of entry across the epithelium and increase the efficiency of delivery will first be discussed. However, directing vaccines to the appropriate location is not sufficient to ensure optimal vaccination effect. The architecture, size, and surface chemistry of particles are of prime importance and can be manipulated to influence the intensity and type of immune responses. Physicochemical properties of lipid-based particles, Ag incorporation, mucoadhesion, and association with adjuvants will be discussed next. Examples of mucosal application of such formulations in animal models and their outcome will then be presented. Finally, an overview of the current evaluation of lipid particles and open challenges of mucosal vaccination in humans will be considered.

## Vaccine Sampling at Mucosal Surfaces and the Selection of the Route of Administration

When applying a vaccine formulation *via* any delivery route, the anatomical, functional, and immunological characteristics of the different tissues have to be considered (13). Indeed, the structure and spatial organization of the tissues, the presence of mucus and mechanisms to eliminate deposited materials/particles (e.g., peristaltism in the intestine and physical discharge in the respiratory tract), the pro-tolerogenic environment of mucosae and the presence and localization of particular immune cell subsets, especially dendritic cells (DCs), all impact on the outcome of vaccine administration (16). In addition, safety issues have to be considered. In this section, we will present the characteristics of the mucosal immune system in relationship with vaccination, the different mucosal administration routes, as well as the strategies under evaluation to increase the efficiency of vaccine delivery.

### The Mucosal Immune System and Mucosal Vaccination

Mucosal surfaces are continuously exposed to, and challenged by, numerous environmental Ags present, for example, in food, air, or derived from pathogenic or commensal microorganisms in the lumen. On top of the epithelial barrier covered by mucus and the secretion of anti-microbial peptides, a specialized and complex immune network, called mucosa-associated lymphoid tissues, is involved in immunosurveillance of mucosal tissues (17). Lymphoid cells and effector molecules, such as secretory IgA (SIgA), the chief Ab molecule operating at mucosal surfaces (18), cytokines, and chemokines, tightly orchestrate protection against infections and maintenance of tolerance toward endogenous unharmful microorganisms. Sampling for such agents and their delivery to immune cells underneath the epithelial layer takes place *via* direct uptake by DCs within the epithelium (19–21) or across specialized epithelial cells named microfold (M) cells that are responsible for the selective transport of macromolecules, particulate Ags, and microorganisms (22, 23). Internalization *via* M cells occurs through different mechanisms (clathrin-coated endocytosis, actin-dependent phagocytosis, or macropinocytosis) depending on the nature of the Ags. M cells are present in (a) the follicle-associated epithelium that separates the intestinal lumen (apical side) from underlying immune cells (basolateral side) in Peyer’s patches (PPs), (b) in intestinal isolated lymphoid follicles, (c) in nasopharynx-associated lymphoid tissues (NALT), and (d) in bronchial-associated lymphoid tissues (BALT) (24). Such structures are composed of innate and adaptive immune cells, including functionally different DC subsets, T, and B cells. DCs integrate signals derived from the sensing of the luminal environment, and release soluble factors, such as cytokines and chemokines, to orchestrate the generation of tightly controlled mucosal immunity locally or after migration in regional lymph nodes (LNs) (7). In addition, paracellular and transcellular uptake of macromolecules and small particles across the epithelia lead to their uptake by APCs outside inductive sites for induction of local immunity *via* regional LNs. By contrast, in the urogenital tract and in the oral cavity, the stratified epithelium does not contain M cells and sampling occurs by DCs interspersed within the tissue leading to induction of immune responses exclusively in draining LNs (15, 25, 26).

Upon encounter with microorganisms or vaccine formulations, mucosal DCs in combination with neighboring epithelial cells control the expression of specific homing receptors on primed lymphoid cells and modulate the type of ensuing immune response (8, 9, 27). Such imprinting relies on the expression of site-specific integrins and chemokine receptors by B and T cells and allows their transit *via* the lymph and through the blood to migrate to different mucosal sites. Recirculation of lymphocytes to the gut requires expression of α4β7 and CCR9, whereas migration to the airways, the oral cavity, and the reproductive tract relies on L-selectin and CCR10. In the case of pathogenic infections, danger signals generated by the sensing of microorganisms switch immune responses toward an effector type of response relying on both humoral and cellular arms to eliminate the infection (28). Immune exclusion and neutralization by SIgA, as well as production of Th1- or Th17-type cytokines that activate phagocytes and induction of cytotoxic T cells, all contribute to the protection of mucosal surfaces (29, 30). Therefore, the major aim of vaccines would be to elicit specific B and T cell responses at the relevant sites to induce specific SIgA that provide a first line of protection against invading pathogens, together with appropriate cellular immune responses to eliminate both the pathogen and pathogen-infected cells. For example, requirements for proper B cell isotype-switching and the generation of IgA responses include mainly the production of TGF-β, IL-6, retinoic acid, and IL-21 by PPs’ cells, together with the CD40–CD40L interaction between T follicular helper cells and B cells (31). Thus, vaccine formulations for mucosal application have to be designed to best induce such immunological environment.

### Administration Routes for Mucosal Vaccination

Each route of mucosal administration has its own characteristics and a balance between the pros and cons for each vaccine has to be considered, taking into account the pathogen to fight against and the formulation to be delivered. However, no standardized studies are available to directly compare safety, profile of induced immune responses, and efficiency of protection. In this section, we will consider the different mucosal administration routes. Oral and nasal/pulmonary are the most studied ones, but sublingual is now recognized as a promising way of vaccination. Vaginal and rectal delivery have also been studied, but more scarcely. Even though the most powerful response is usually elicited in the local inductive and adjacent tissues, the common mucosal immune system predicts that homing to distant mucosal tissues is possible (27). However, a certain degree of compartimentalization does not allow imprinted cells to migrate to every mucosal sites. Such flexibility allows to select for the most appropriate route of vaccination to induce protective immune responses at the desired site. Of note, some recent works demonstrated that transcutaneous immunizations have the potency to promote the induction of immune responses with the ability to traffic to the gut and airways, although up to now with low consistency (32). However, this aspect will not be covered by this review and has been described elsewhere (33, 34).

Nasal administration represents a promising route for mucosal vaccination (35), because nasal tissues display a relatively large surface for Ag absorption covered with only a thin layer of mucus, and are highly vascularized. It does not require the delivery of high Ag doses (e.g., as compared to oral administration), is non-invasive and easily accessible for self-administration. Nasal vaccination allows the generation of a broad range of Ab and T cell responses at different mucosal sites, such as the upper (preferentially) and lower airway mucosae, the local secretions, the salivary glands, and the urogenital tract. It also elicits concomitant robust systemic immunity (15). However, in the nasal environment, the presence of proteases and the local pH, together with a relatively high mucoscillary clearance rate, may impact on the vaccine integrity and retention time, thus affecting the generation of immune responses. The major drawback concerns the safety of nasal administration, as physiological function such as smell perception might be altered by vaccine-induced inflammation and the close relationship with the brain might promote health problems. Thus, every vaccine candidate has to be evaluated carefully for safety. Sometimes achieved by nasal delivery or directly targeted, pulmonary immunization allows vaccine formulation to directly access the respiratory tract which is of interest due to its high permeability, its large surface area and the high density of APCs (alveolar macrophages, DCs, and B cells). This route of administration preferentially induces immune responses in the lower airways and has interestingly been shown to promote cellular and humoral responses in the gut. However, efficient delivery in the lung is not an easy task. Delivery *via* the nasal and pulmonary routes does not necessarily lead to similar outcomes: for example, pulmonary vaccination was shown to be more effective than its nasal counterpart at protecting against *Mycobacterium tuberculosis* infection, because different immune mechanisms were involved after one or the other administration route (36–38). Indeed, elevated levels of SIgA were produced in the lung after pulmonary vaccination, with equivalent responses observed in the nasal passage. In addition, IFN-γ production in the lung following pulmonary vaccination was important to fight against *M. tuberculosis*, whereas there was apparently no role for this cytokine in the nasal environment.

Oral administration represents an interesting strategy in terms of ease of delivery, patient compliance, and safety (39). However, due to the intrinsic high dilution of vaccine formulations and the harsh environment of the digestive tract, substantial amounts of Ags have to be administered. Indeed, the extremely low pH in the stomach, proteolytic enzymes and bile salts in the intestine, the presence of relatively thick one-layered mucus, and the overall low permeability of the intestine greatly affect the integrity and delivery of applied Ags. In this context, oral vaccines are likely to be more efficient if repeated doses are given, provided that adjuvants are incorporated to avoid tolerance induction (13). Oral administration is the most efficient delivery route to achieve induction of gut immunity, which is of high importance to fight against the large burden of enteropathogenic infections worldwide. Induction of immune responses in the colon, stomach, mammary, and salivary glands, as well as systemically, also takes place, but with limited robustness (15).

Sublingual immunization generates immune responses with similar profile and mucosal tropism as nasal delivery, i.e., vigorous and broadly disseminating mucosal and systemic IgA and IgG, as well as helper and cytotoxic T cell, responses (40), without many side effects (41), and formulation concerns associated with nasal or oral immunization (42). It is also easily accessible for self-administration. It has been shown to induce immune responses after administration of soluble Ags, particulate Ags, live/killed bacteria, and viruses (40). Sublingual delivery is interesting because the oral cavity has a milder environment that may not degrade vaccine components, and may, thus, not require large amounts of Ags. As an example, higher Ab responses were obtained in mice after sublingual, as compared to oral, administration with about 10–50 times less Ag applied (43). One limiting factor is the absence of Ag-sampling M cells in the oral cavity lined by a stratified epithelium and the relatively low number of DCs in the upper layer of oral tissues. However, vaccine formulations can be taken up by lingual tonsils for delivery into regional LNs and Langerhans cells in the oral epithelium have been shown to act as potent inducers of immunity (15). Several delivery systems (microneedles, liposomes, inactivated microorganisms) and adjuvants [toll-like receptor (TLR) ligands, cholera toxin (CT), mutants of heat labile toxin (LT) and CT] have been evaluated for sublingual vaccination and protective Th1-type responses in the lung, genital tract, and the gut, together with SIgA in the saliva, intestinal, and vaginal washes, have been obtained with different vaccine formulations (42–45).

Vaginal immunization elicits immune responses in the genital tissues and secretions, but is not efficient to induce systemic immunity. Despite relatively low pH, the vagina is a mild environment that does not impair Ag integrity and, thus, allows to limit the amount of Ag to be delivered (25). However, the presence of a stratified epithelium and the absence of inductive sites imply that induction of vaccine response *via* vaginal delivery requires specific adjuvanted formulations and DC subsets (46). In addition, the changes occurring in term of immunological functions during the estrous cycle complicate such immunization (47–49). Additional studies are required to better understand mucosal immunity in the urogenital tract and define specific requirements for vaccine formulations. Rectal immunization is able to induce potent immune responses in the small intestine and the colon, but not efficiently in the systemic compartment (15). Only limited studies are available to fully appreciate the potential of such an administration route for mucosal vaccination.

### Targeted Delivery of Vaccine Ags

Not only do vaccine formulations have to resist the deleterious environment of some mucosal surfaces, but they also have to face an additional hurdle that is to cross the epithelium to gain access to underlying APCs. In this context, targeting the relatively low number of portals of entry at inducing sites, e.g., M cells that represent 1% of epithelial cells (5–10% of enterocytes within the follicular-associated epithelium), or DCs spread within the epithelium is an asset for efficient vaccination. DC targeting by the mean of C-type lectin receptors (DEC205, DC-SIGN, mannose receptor) or specific Abs directed against DC markers has proven to be an efficient strategy to improve the potency of parenteral vaccination (50). Similar strategies have been developed for mucosal vaccination, such as targeting of Langerin on DCs of the oral cavity, the esophagus or the vaginal mucosa (51). FcRn expressed by airway and gut epithelial cells (52, 53), as well as some DC subsets (54), has also been demonstrated as an efficient strategy to deliver IgG-based complexes across the epithelium and to underlying DCs. Such an approach efficiently led to the induction of both CD4 and CD8 T cell effector responses (55–57). Galactosyl ceramide may function as a targeting moiety in the intestine, the rectum, and the endocervical mucosa (58), and the ganglioside GM1 molecule can be targeted by a specific peptide developed by phage display (59). As far as M cells are concerned, specific delivery can be achieved *via* different strategies: the tight junction molecule claudin-4 (60), the bacterial FimH receptor GlycoProtein-2 (61, 62), the complement C5a receptor and its ligand Co1 (63, 64), a M-cell-specific peptide referred to as CKS9 (65), or the unique glycosylation pattern involving α-1-fucose; for the later, the use of Ulex Europaeus Lectin-1, or a specific monoclonal Ab have been successfully demonstrated (66–68). However, in humans, the lack of expression of this particular sugar moiety on M cells (69), together with the extra-M cell expression of GP-2 (70), precludes the use of such strategies for specific targeting purposes. By contrast, a promising approach consists in coupling vaccine Ags with SIgA in order to induce M-cell-specific retrotransport across the epithelium and DC targeting *via* Dectin-1 in both mice and humans (71). This Ab molecule can potentially serve as a cargo for the controlled delivery of the associated payload as this occurs naturally with microorganisms sampled from the mucosal lumen (72). An additional advantage is the resistance of SIgA to protease degradation and its ability to anchor in mucus (18), two features that may improve both stability and retention time of the associated Ags.

## Formulation Considerations

Particulated Ags have been designed to mimic the shape, size, and antigenic display of pathogens with the aim of improving vaccine efficiency (73). Ags associated with micro-/nanoparticles display increased depot effect upon administration, are better protected from degradation, and are more efficiently taken up by APCs and presented to B and T cells than soluble Ags (74). An important number of studies have evaluated the effect of particle properties (type of material, size and charge, Ag incorporation, or association of adjuvants) on the profile and strength of induced Ab and T cell responses. When composed of natural lipids, lipid-based particles have the advantage of being biocompatible. In addition, they are very flexible in terms of formulation, implying that lipid exchange within the particle shell is achievable, leading to modulation of their physico-chemical properties. This is of importance in the biological environment because all these parameters will influence the stability and immunological consequence of delivered particles. However, minor changes in the composition of the particle may impact on its efficiency and protective ability, meaning that any formulation needs to be individually evaluated *in vivo*.

### Size and Charge of Particles

The size of particulate Ags has an impact on the type of immune responses that are generated because it influences the mechanism of uptake by APCs. Indeed, receptor-mediated endocytosis, pinocytosis, macropinocytosis, or phagocytosis, all lead to different ways of trafficking within the cells and, therefore, induce preferentially presentation *via* the MHC I or MHC II pathway for CD8 or CD4 T cell priming, respectively (75). Small particles (up to 200 nm) are sensed as viruses and are taken up by receptor-mediated endocytosis leading to predominant T cell responses, whereas larger particles (more than 500 nm) are taken up *via* micropinocytosis or phagocytosis to preferentially induce Ab responses (76). Similar size-dependent uptake by M cells or enterocytes takes place, leading to differential sampling of the particulated Ags. Other studies demonstrated that vesicles smaller than 250 nm induced a balance toward Th2-type of responses, whereas the opposite was observed for larger vesicles (77–79). Moreover, in the context of mucosal delivery, the size of the particles influences the tissue localization and the diffusion across the mucus. Following nasal administration, small particles are better transported across the nasal mucosa, whereas larger ones are better deposited in the respiratory tract to be taken up by alveolar macrophages (80, 81). In order to get access to the epithelium, both viscosity and pore size of mucus can impact on the penetration of vaccine components. Apparently, the average size of pores in the mucus is in between 200 and 500 nm, e.g., in the cervicovaginal and small intestinal mucus. This suggests that particles smaller than this cutoff freely diffuse across the mucus, whereas larger ones take more time to reach the epithelium or possibly never reach it (82, 83). One major point to be considered is that correlation between size and immunogenicity is difficult to strictly assess for lipid-based particles, because homogeneous and monodispersed preparations have been challenging to obtain, and when feasible, such preparations require technical issues that may dramatically increase the cost of vaccine formulations.

Diffusion across the mucus is not only governed by size of particulated Ags or mucus pores but also by chemical characteristics such as the surface charge of particles. Hydrophobic and electrostatic interactions mediated by particles aggregate mucus microstructure and impede diffusion of vaccines, while hydrophilic and neutral vaccine formulations promote mucus penetration (23). Mucoadhesion is promoted by positively charged particles that interact with negatively charged mucus. For example, electrostatic interactions between cationic lipids and the nasal mucosa promote enhanced contact time with the tissue, higher local concentration, and thus improved penetration of liposomes (84). Similarly, cationic particles better interact with negatively charged cell membranes, such as those of M cells and enterocytes, therefore limiting vaccine clearance and improving sampling *via* endocytosis or membrane fusion (85, 86); it also improves the uptake by DCs (87). However, cationic particles may have charge-dependent cytotoxicity against target cells; therefore, the density of cationic lipids within the particle shell has to be carefully defined and a tight balance between strong adhesion and safety has to be achieved (23). Interestingly, it seems that the presence of the mucus limits cytotoxicity (85), meaning that cationic particles keep their validity for mucosal vaccination.

### Incorporation of Ag

There are different ways of associating Ags to lipid-based delivery systems and the choice depends mainly on the administration route and the nature of the Ags (88, 89). For oral administration, encapsulation seems favorable in terms of ensuing immune responses, because it prevents rapid degradation of the Ag within the gastrointestinal environment and, hence, increases its half-life (90). Encapsulation of Ags is relatively easy to perform during the manufacture process, but this may alter antigenic structures. By contrast, maintenance of the integrity of the Ags is less affected by the nasal route, suggesting that surface association *via* charge interaction is sufficient. Such an approach is technically not demanding, owing that opposite charges of either the particles or the Ags favors it (90). Alternatively, covalent binding at the surface of particles is achievable, although more complicated to perform; this precludes the undesired release of the payload that may occur within the tissue environment. In terms of immune response induction, encapsulation within liposomes preferentially induces IgG production, whereas surface display of the Ag induces both IgM and IgG responses (91), with elevated levels (92). In addition, Ag density at the surface of particles, as well as the Ag-to-lipid ratio, has been documented to influence the elicited immune responses following immunization (93, 94). This may suggest that both encapsulation and surface location of the Ag within the same formulation would promote optimal induction of T cell and B cell responses.

Apart from protein Ags, plasmid DNA coding for pathogen-derived Ags have been evaluated for vaccination (95, 96). Such strategy has an established record of efficacy in preclinical studies and can be safely used in humans, even in immunocompromised individuals. However, based on results obtained in the field of veterinary vaccination, naked DNA induces only weak immune responses. In order to improve immunogenicity, association of DNA with cationic liposomes leads to increased uptake by target cells and delivery into the nucleus. DNA immunization through the nasal or the oral route can effectively induce protective humoral and cellular immunity at related mucosal surfaces, but necessitates association with cationic delivery systems, presumably to increase mucus penetration, to reduce mucociliary clearance, and to improve permeation across the epithelium (97, 98). DNA sequences such as the canonical CpG motifs have been shown to display immunostimulatory properties. In a similar way, messenger RNA-based vaccines, when approprietely protected from ribonucleases are translated in the cytoplasm and do not require nuclear transport (99). This has been mainly evaluated with cationic lipid-based vesicles in the context of cancer immunotherapy, and the efficient nasal application of particle-associated mRNA has been demonstrated (100).

### Mucoadhesive Properties

Upon mucosal administration, vaccine formulations are diluted in mucosal fluids and have to face bulk flow, leading to limited retention time and suboptimal access to the epithelium for sampling. Such deleterious effects can be compensated by incorporation of mucoadhesive and mucus-penetrating components. In this case, the surface structure of lipid-based particles has to be carefully designed to obtain an adequate balance between strong adhesion and mucus penetration. Some possible strategies are described below. The first one consists in incorporating polyethylene glycol (PEG) at the surface of particles. PEG has originally been used for systemic administration in order to avoid adsorption of plasma proteins and the formation of a corona that may mask targeting ligands, adjuvants, or Ags at the surface of particles (101). In addition, the presence of PEG increases the stability upon administration. A similar stabilization effect has been reported in the case of oral or sublingual delivery of liposomes (93, 102, 103). PEG is a hydrophilic component that has been reported to help particles to penetrate the nasal mucosa by preventing aggregation and thus facilitating diffusion across the mucosal barrier. Moreover, it can form hydrogen bonds with mucus leading to mucoadhesion, but also helps diffusion across the mucus; indeed, such intriguing bifunctionality has been correlated with the molecular weight of PEG. High molecular weight polymers (>PEG5000) are preferentially mucoadhesive whereas lower ones (PEG2000) better diffuse within the mucus (103–105). An additional non-negligible advantage of PEG is that it provides cryopreservative functions during particle manufacture.

The second strategy is to associate with micro-/nanoparticles some mucoadhesive components, such as chitosan (deacetylated chitin), alginate, polyvinyl alcohol, hyaluronan, or cellulose derivatives that all boost particle-based vaccination (106–108). Addition of bioadhesive components (xanthan gum or tramella) within formulations helps to increase the viscosity of the vaccine and, thus, the retention time at mucosal surfaces. The most studied mucoadhesive molecule is chitosan, whose relevant properties for mucosal vaccination are as follows: (1) it is a positively charged molecule that can interact with negatively charged mucus to improve adhesion; (2) it is a permeation enhancer due to its ability to transiently open epithelial tight junctions and, thus, improve Ag sampling (109); and (3) it has adjuvant properties, promoting induction of IFN-γ, IgG, and SIgA (110). Chitosan has been explored for delivery *via* oral, nasal, and pulmonary routes in association with liposomes leading to increased stability and mucoadhesion for absorption by mucosal surfaces (111). Interestingly, it did not demonstrate detrimental effects toward mucosal tissues (112).

### Incorporation of Adjuvants

Several adjuvants have been evaluated during the last decades for mucosal vaccination (113). Essential properties of the ideal adjuvant include the following: to be effective with low-dose Ag; to be suitable with many different Ags; to be effective enough to reduce the number of vaccine administrations; to be able to induce long-term immune responses; and to display limited or absent toxicity. Innate immune triggers have been used as adjuvants as they have the capacity to elicit pro-inflammatory responses to recruit phagocytes, to enhance Ag presentation by APCs, and to activate APCs in order to generate the adequate environment for efficient priming of adaptive immunity. Studies in animals have demonstrated an important adjuvant effect of the *Vibrio cholerae* endotoxin CT and *Escherichia coli* LT, ensuring enhanced Ag permeation through the epithelium, enhanced targeting of M cells, increased Ag presentation by DCs and improved activation of DCs (114); a direct effect on B and T cells has additionally been reported (15). However, such adjuvants are inadequate for human use because of their toxicity and unacceptable side effects, as for example: induction of deleterious inflammatory response leading to altered function of olfactory nerves or to Bell’s palsy after nasal administration, and diarrhea symptoms after oral administration (115, 116). This has oriented research toward the generation of less toxic derivatives engineered by introduction of mutations in the A subunit of CT and LT (117–120). The most promising derivative is the double mutant LT (R192G/L211A, dmLT) that has no demonstrated side effects in animal application while retaining important adjuvant activity after oral or sublingual administration (121–123). Similar mutations R192G/L211A applied to CT similarly reduced its toxicity, although to a level still not acceptable for human use. Introduction of additional mutations within the amino acid 189–197 stretch recently demonstrated more safety with an ability to induce both Ab and T cell responses close to that of the non-mutated CT following nasal, oral, and sublingual vaccinations (124). An alternative approach is the use of the B subunit of LT or CT only. LTB and CTB are not very efficient *via* the oral route, however, nasal administration demonstrated some efficiency when the Ag was physically linked to the adjuvant resulting in increased uptake across the epithelium and by DCs (15). Fusion protein obtained by association of the A1 subunit of CT and *S. aureus* protein A derivative (CTA1-DD) proved efficient at boosting B cell responses after nasal administration (125). In another report, edema toxin from *Bacillus anthracis* and diphtheria toxoid within lipidic particles have been evaluated for nasal administration and showed efficient induction of immune responses leading to reduced bacterial load after pathogen challenge (126).

In parallel, evaluation of immunostimulatory molecules active for parenteral administration, such as TLR ligands, have been performed (113). CpG oligodeoxynucleotides (CpG), monophosphoryl lipid A (MPLA), and flagellin that are ligands for TLR-9, TLR-4, and TLR-5, respectively, have been administered orally or intranasally and demonstrated immunostimulatory properties for mucosal immune responses, including induction of SIgA (127–131). Pulmonary delivery of a *M. tuberculosis*-derived Ag together with CpG or MPLA promoted the generation of IFN-γ production in the lung; MPLA was more potent to induce IL-17 production and to decrease the bacterial load following challenge (132). Flagellin, expressed by different pathogenic bacteria, can indirectly stimulate local DCs following nasal delivery, and induce mucosal IgA responses and protection upon Influenza vaccine administration (133, 134). Alternatively, trehalose dibehenate (TDB), a synthetic analog of a *M. tuberculosis* cord factor known to interact with Mincle and promote Th1/Th17-type of responses (135), has also been shown to be effective by the nasal route (136). Saponin QS21 also demonstrated potent adjuvant effect when nasally administered (128, 129). In addition, STING ligands 3′3′-cGAMP, c-di-AMP, and c-di-GMP have been efficiently delivered *via* the nasal or the sublingual route to elicit Th1/Th17 responses and high-affinity SIgA (137). Activation of NKT cells by administration of α-galactosylceramide is also of interest for nasal, oral, and sublingual vaccination due to its ability to enhance immunogenicity of different mucosal vaccine formulations (138–140). All these adjuvants can be incorporated within lipid-based particles or associated at their surface depending on the localization of their cognate receptor in target cells. This has been successfully achieved and resulted in improved uptake by and activation of DCs (141). Moreover, cationic lipids *per se* have been shown to directly activate APCs (142).

## Lipid-Based Particles for Mucosal Vaccination

**Figure 1:**
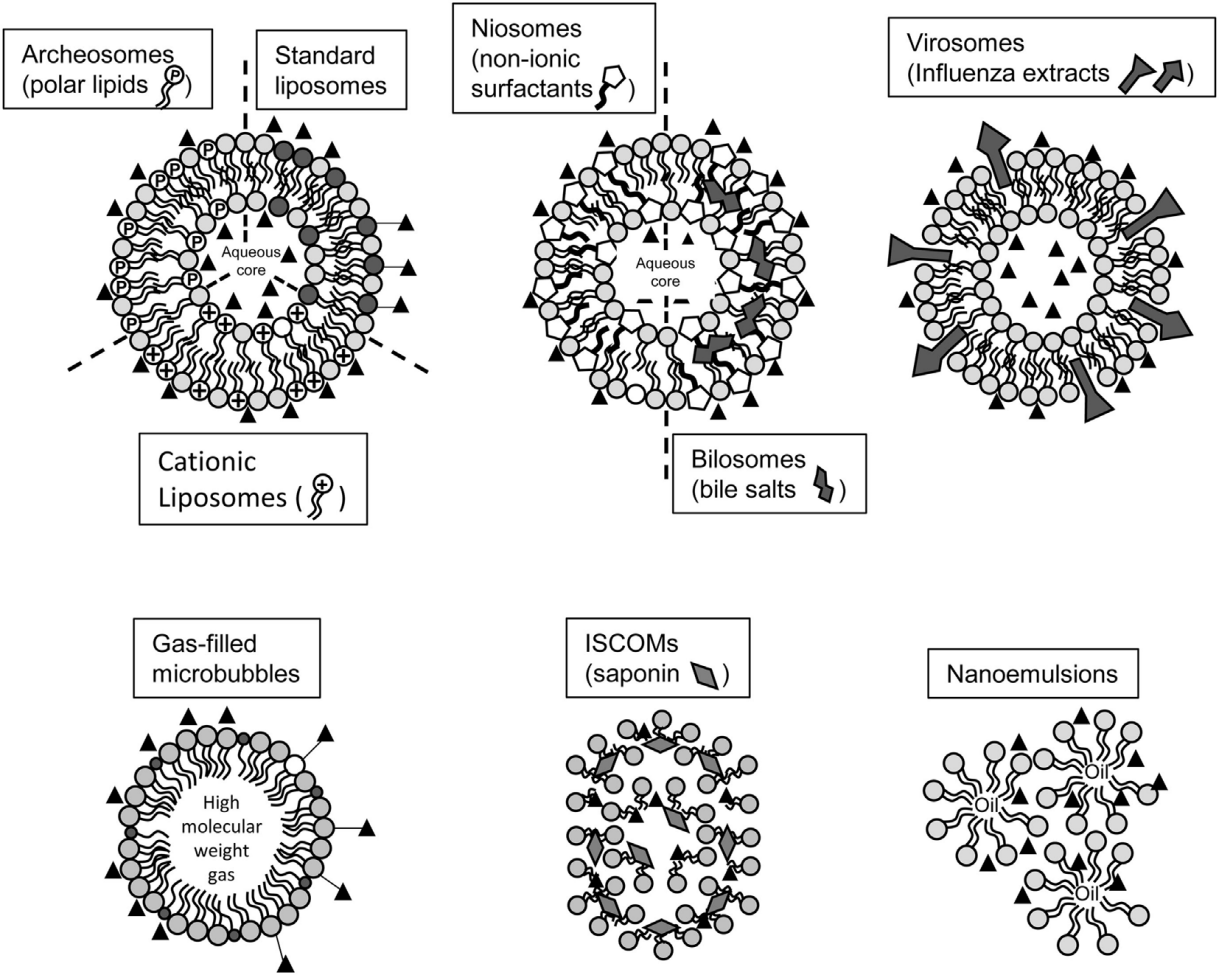
Schematic representation of lipid-based particles evaluated for mucosal vaccination. Liposomes can be tailored to incorporate particular lipids (cationic lipids, polar lipids from Archea), Influenza extracts or non-ionic surfactants in order to improve stability and immunogenicity of vesicles. Other lipid-based structure incorporating saponin, entrapping inert high molecular gas, or composed of emulsions has been developed for vaccination purposes. Black triangles represent Ags. They can be entrapped in aqueous cores, entrapped in hybrophobic parts, associated at the surface of particles through electrostatic interactions, or covalently linked at the surface of particles. Not drawn on scale.

In order to induce efficient and protective immune responses by vaccination, not only the Ags and the adjuvants have to be carefully defined, but also an appropriate delivery system is of prime importance. When aiming at using mucosal routes of administration, they must be designed to resist chemical degradation by low pH, proteolytic enzymes, and the harsh environment of mucosal surfaces. Lipid-based particles represent interesting delivery systems to incorporate Ags and adjuvants, allowing targeted and concentrated delivery of relatively low amounts in tissues, together with limiting toxicity associated with potential spreading of the payload (Figure 1). Many lipid-based particles have been tested in animal models of immunization and/or infection and are reviewed below (Table 1). Advantages, limitations, and necessary refinements for use as effective mucosal vaccine are discussed sequentially.

**Table 1:** Lipid-based formulations evaluated for mucosal vaccination in mouse models.

	Structure	Evaluated mucosal routes	Advantages	Stability	Limitations
Liposomes	Bilayer of phospholipids entrapping an aqueous core	Nasal, oral	Flexibility in lipid composition, ease of Ag/adjuvant incorporation, immunogenicity of cationic liposomes	Relatively low intrinsic stability for storage and after administration	Potent toxicity of cationic lipids (dose-dependent)
Archaeosomes	Liposomes composed of Archaea-derived polar lipids	Nasal, oral	Improved immunogenicity	Improved stability as compared to liposomes	Preparation of Archea lipids
Niosomes, bilosomes	Cholesterol-based liposomes with non-ionic surfactants and bile salts	Oral	Ease of manufacture	Improved stability as compared to liposomes	Low flexibility in lipid composition, low immunogenicity
Virosomes	Liposomes containing lipidic viral extracts	Nasal, sublingual	Immunogenic without addition of adjuvant	Good stability	Purification of Influenza extracts
ISCOMs	Cage-like structure made of cholesterol, phospholipids and Quil A saponin	Nasal, oral, vaginal	Self-adjuvanted due to saponin	Good stability	Difficult to incorporate non-lipidic Ags
Microbubbles	Monolayer of phospholipids/palmitic acid entrapping an inert gas	Nasal, oral	Flexibility in lipid composition	Limited stability upon reconstitution and administration	Difficult to entrap Ags
Emulsions	Oil-in-water nanosized droplets	Nasal, oral	Ease of manufacture, self-adjuvanted	Limited stability after administration	Low protection of Ag structure

### Liposomes

The enormous potential of liposomes for drug delivery has been acknowledged for decades. Indeed, they display features including controlled release, protection from degradation, improved pharmacokinetics, increased circulation time, and targeting to specific tissues (143, 144). They have been progressively adapted for administration of diverse antigenic entities, such as proteins, peptides, and DNA, in order to produce vaccine formulations to fight against several viral and bacterial infections (89). Liposomes are spherical vesicles consisting in unilamellar or multilamellar shell of phospholipid bilayer(s) entrapping an aqueous core and range in size from tens of nanometers to several micrometers in diameter. The amphiphilic nature of phospholipids mediates self-assembling of liposomes in an aqueous environment leading to a bilayer configuration. They can incorporate both hydrophilic molecules encapsulated within the aqueous core and hydrophobic molecules hooked at their surface or inserted within the inner hydrophobic space of the lipid bilayer. Biocompatible neutral and anionic phospholipids, such as phosphatidyl cholines [e.g., distearoylphosphatidylcholine (DSPC)], and cholesterol, are the most commonly used constituents of the shell that ensures proper stability of the structure and improved immunogenicity of the formulation (145, 146). The length and degree of saturation of acyl chains influence both the permeability and the fluidity of the shell, leading to increased or decreased stability. Liposomes are versatile delivery systems that are interesting for vaccination formulations because their physicochemical properties can be modulated by altering their composition in lipids. Among possible modifications, pH titrable lipids to induce controlled release of payloads (147, 148) and synthetic cationic lipids to improve immunogenicity (90) have been generated. In addition, functionalization of liposomes with specific targeting moieties has emerged as a promising strategy to improve delivery. Vesicles bearing the DC-SIGN-specific ligand Lewis x glycan showed increased DC-targeting properties and subsequent activation of T cells, especially when adjuvanted (141) and IgG-coupled liposomes have demonstrated enhanced transmucosal transport in nasal tissues (55).

Cationic liposomes prepared with dioleoyltrimethylammoniumpropane (DOTAP), dimethyldioctadecylammonium bromide (DDA), dimethylaminoethane-carbamoyl (DC)-cholesterol have been successfully evaluated. Nasal administration of DDA-based liposomes induced greater local and vaginal IgA production as compared to vesicles without cationic lipid. Moreover, the incorporation of PEG further increased the observed immune responses (149). Similarly, delivery of cationic liposomes composed of DOTAP and DC-cholesterol *via* the nasal route allowed efficient uptake by DCs in NALT and subsequent induction of specific IgA and T cells in nasal tissues (150). The adjuvant CAF01 is a prime example of efficient cationic liposomes to be used for mucosal vaccination. Incorporation of both DDA and the immunostimulatory molecule TDB, has been evaluated in several animal models of infections with Influenza, *Chlamydia* and *M. tuberculosis* (151). In such context, the presence of the adjuvant had a substantial beneficial effect on immunogenicity (152). Nasal vaccination against Influenza or *Streptococcus pyogenes* with CAF01-based formulations allowed to generate mucosal effector T cell and IgA responses and to protect vaccinated animals (136, 153). Furthermore, preparation of liposomes with the cationic lipid ceramide carbamoylspermine efficiently stimulated systemic and mucosal immunity following intranasal administration (154). The use of cationic preparations is also an interesting approach for alternative forms of antigenic entities, as liposomes incorporating DOTAP and a plasmid DNA coding for a mycobacterial heat-shock protein given nasally induced local mucosal immune responses able to reduce *M. tuberculosis* load in the lung (155). Overall, liposome-based vaccination *via* the nasal route leads to the induction of robust immune responses whatever the nature of the Ag and its mode of incorporation. Thus, fine-tuning modulation of the profile of vaccine-elicited responses appears to depend on the composition of the formulation, including the type of lipids and/or the presence of adjuvants. Interestingly, most liposomal preparations seem to be well tolerated, inducing only limited inflammatory responses, irritation, sneezing, or burning syndroms. Oral administration of liposomes has been documented, but its stability in the gastrointestinal tract remains the main concern. As already discussed in Section “Mucoadhesive Properties,” promising approaches can be envisaged to improve the stability of liposomal preparations. Incorporation of mannose, chitosan, and PEG are all possible scenarios resulting in reinforced stability, better targeted delivery across the epithelium and to APCs, and improved immunogenicity. Stabilization of liposomes with layer-by-layer deposition of polyelectrolytes also increased the generation of Ab and T cell responses in mucosal tissues (156). The administration of multilamellar preparations is an alternative strategy. Finally, as discussed in Section “Administration Routes for Mucosal Vaccination,” vaccination *via* the sublingual route is a promising development that requires to be further evaluated for liposomal preparations in the context of infectious diseases. For example, PEG-modified liposomes incorporating Influenza-derived Ags, together with the TLR-4 agonist CRX-601 as adjuvant, were effective at eliciting elevated levels of serum neutralizing Abs and mucosal IgA (103).

### Liposome-Derivatives

Derivatives of liposomes have been explored to circumvent some of the drawbacks associated with liposomes and to improve their efficiency. For example, association of non-ionic surfactants with cholesterol or its derivatives to generate a structure called niosomes has allowed to increase the stability of the bilayer vesicles by preventing oxidation of the lipids (157). Addition of mannan at the surface of niosomes further increased the stability of the vesicles and helped to target specific receptors on APCs following oral administration. Vaccination with niosomes incorporating plasmid DNA coding for an Hepatitis B Ag induced SIgA production in the salivary and intestinal fluids, together with systemic Th1-type T cell responses (158). Moreover, incorporation of bile salts within niosome structures (bilosomes) has been shown to increase the stability of the vesicles and thus to improve oral delivery of peptides and proteins to the gut immune system (159, 160). Bile salts, such as deoxycholic acid or taurocholic acid, are amphiphatic molecules that can be easily incorporated within lipid bilayers and can promote the passage of lipophilic components across cell membranes (161). Thus, bilosomes have the ability to reinforce the biovailability of associated Ags mainly for oral vaccination (162). Different examples of bilosome application have been reported in association with Hepatitis B-derived Ags and Tetanus toxoid. In this context, induction of SIgA in mucosal secretions and IgA-positive plasma cells were observed (162–164) and showed elevated responses as compared to parenteral injection or use of niosomes without bile salts. An alternative approach is the inclusion of polar lipids with fully saturated isoprenoid chains extracted from Archaea to generate vesicles called archaeosomes or archaeal lipid mucosal vaccine adjuvant and delivery (AMVAD) (165). They have been shown to induce robust long-lasting protective Ab and T cell responses, including cytotoxic T lymphocytes responses after systemic injection (166). Advantages of such structures for mucosal vaccination comprise increased pH-dependent and thermal stability due to prevention of lipid oxidation and resistance to phospholipases and bile salts. In this context, mice immunized by the nasal route demonstrated sustained robust local and distant IgA responses in mucosal fluids, strong systemic IgG responses, and T cell responses (167). In addition, nasal vaccination with archaeosomes and cell-free extracts of *Francisella tularensis* led to reduced bacterial burden in the lung and spleen in a mouse model of tularemia (168). Oral immunization with archaeosomes is possible as well, although with higher amounts of Ags. Improved stability and retention time of such vesicles has been observed in the intestine, leading to potent IgG and IgA production (169). One non-negligible drawback of this approach is the access to achaeal polar lipids, as the purification from Archaea is a relatively demanding process. Nevertheless, production of synthetic polar lipid structures is under development.

### Virosomes

Virosomes are a special category of liposomes, where part of the lipid content is derived from viral components that self-assemble into an organized three-dimensional structure that mimics the antigenic structure of the original virus (170). Interestingly, they have been demonstrated to be immunogenic without further addition of adjuvants (171), although addition of immunopotentiating agents further improves their vaccine efficiency (172). Originally called immunopotentiating reconstituted Influenza virosomes, they harbor hemagglutinin and neuraminidase proteins from Influenza virus. These proteins target sialic acid on cell membranes, leading to fusion between the target cell and virosomes, followed by intracellular delivery of their payload. They exhibit similar flexibility and advantages as standard liposomes; however, the process to extract all the necessary components from Influenza virus is relatively complex. Virosomes have been mainly investigated for parenteral vaccination, but reports on their use for mucosal administration exist. They have been used as prime-boost vaccination strategy in a simian model of HIV infection, where intramuscular injections have been followed by nasal administration. It induced full protection against vaginal simian-HIV challenge that was correlated with the presence of mucosal IgA and IgG with blocking activity against virus transcytosis and neutralizing/Ab-dependent cellular cytotoxicity properties, respectively (173). In mice, nasal or sublingual administrations of adjuvanted virosomes were able to protect against Influenza and respiratory syncytial virus infections by promoting mucosal and systemic Ab responses, together with Th1-type cellular responses (174–176).

### Gas-Filled Microbubbles

Gas-filled microbubbles are microsized spherical structures composed of a lipidic, denatured protein-based, or crosslinked polymer shell generally entrapping inert high molecular weight gases to ensure resistance to pressure once administered (177). Due to their strong echogenicity in presence of low ultrasound intensities, they are currently used for human application as intravenously delivered echo-contrast agents to more precisely visualize for example angiogenesis in malignant tumors, left ventricular opacification, and myocardial perfusion. In addition, cavitation induced by higher ultrasound application leads to the transient nonlethal permeability of the surrounding tissue (e.g., vascular barriers or cell membrane) allowing enhanced local on-demand extravasation and bioavailabilty of microbubble-associated payload (178). In the last decades, such a process, known as sonoporation, has received important attention in order to improve delivery of a wide range of therapeutic molecules, including chemotherapeutic agents, siRNA, miRNA, oligonucleotides, or plasmid DNA, to tumor or immune cells (177, 179). Typically, sonoporation has been used for improved delivery of Ags into DCs with the aim of boosting immune responses (180). Interestingly, lipid-based microbubbles can be taken up by APCs and deliver intracellularly their antigenic payload without ultrasound application, leading to processing and presentation of the Ag to responsive T cells (181, 182). Furthermore, microbubble-associated Ags can be injected parenterally as a vaccine formulation to elicit potent and long-lasting immune responses against systemic bacterial infection (183, 184). Lipid-based microbubbles are usually composed of phospholipids (e.g., DSPC) and palmitic acid, but tailored formulations can be prepared by incorporation of cationic lipids in their shell in order to better associate DNA (179). In addition, to improve the specificity of imaging and drug delivery, microbubbles can be targeted to particular tissues by linking cell-specific ligands or Abs has been developed at their surface (185, 186). Such aspects are of interest for mucosal vaccination using targeting strategies as discussed in Section “Targeted Delivery of Vaccine Ags.” Moreover, adjuvants can be associated with microbubbles, which results in enhanced immunogenicity of the vaccine preparations. As an example, nasal delivery of α-galactosylceramide-adjuvanted microbubbles displaying the *Salmonella*-derived SseB Ag at their surface were able to induce potent IgA, IgM, and IgG humoral responses in the gut, which were associated with a Th1-/Th17-type cellular response. This resulted in a significant decrease in local and systemic bacterial load following oral infection with *Salmonella enterica* Typhimurium in prophylactically vaccinated mice; such effect was more potent than parenteral injection of the same microbubble formulation (140). Despite so far limited induction of local immune responses after oral administration, improvement of microbubble formulations may lead to enhanced immunogenicity. Moreover, sublingual administration remains to be tested owing to its valuable advantages in the context of mucosal vaccination. In recent years, nanosized bubbles have been developed that showed increased stability and extravasation following systemic administration, suggesting that such derivatives might be even more suitable for vaccination purposes (187).

### Immune Stimulating Complexes

Immune stimulating complexes (ISCOMs) are negatively charged self-assembling pentagonal dodecahedrons cage-like rigid structures with a size of 30–40 nm. They can form spontaneously after mixing Ags with cholesterol, phospholipids (usually phosphatidylethanolamine and phosphatidylcholine), and the saponin Quil A extracted from the bark of *Quillaja saponaria* Molina tree. Interestingly, such formulation allows to reduce the toxicity associated with saponin administration, while retaining its adjuvant activity (188, 189). Proteins or glycoproteins that are normally anchored by a hydrophobic transmembrane sequence into the cell membrane can be incorporated as such. Non-amphipatic proteins or peptides have to be modified by attachment of a lipid tail (e.g., palmitic acid). Immunization with ISCOMs induced both Th1-type humoral and cellular responses, including cytotoxic T lymphocytes that are important to fight against intracellular pathogens (190). Several studies have reported potent induction of mucosal immune responses, including robust IgA production in nasal washes and the lung, after nasal/pulmonary vaccination with ISCOMs harboring antigenic entities from Influenza virus (191–194), respiratory syncitial virus (195), Hepatitis B virus (196) and measles (197). Protective efficacy was observed as well after vaccination with an Influenza subunit vaccine composed of ISCOMs (198), adjuvanted ISCOM-based anti-*M. tuberculosis* and anti-Influenza vaccines (199, 200) and *Helicobacter pylori*-Ags delivered *via* ISCOMs (201). In some cases, such immunization proved more efficient than parenteral injection (196, 202). Production of ISCOMs with an alternative saponin, derived from *Quilaja brasiliensis*, also allowed to induce mucosal local and distant IgA production after nasal delivery of an OVA-based vaccine (203). In addition, incorporation of DNA plasmid within the ISCOM matrix elicited potent anti-*Haemophilus influenzae* cellular and Ab responses in the nasopharynx of nasally immunized animals (204). Oral administration of ISCOM-based vaccines has been evaluated (205, 206); however, it seems that the generation of intestinal IgA responses was limited (207). Although ISCOMs are self-adjuvanted delivery systems, incorporation of the adjuvant CTA1-DD within the structure allowed to induce robust mucosal IgA production and T cell proliferation, together with systemic responses, after nasal administration (208). Such an approach has also been evaluated *via* oral delivery. Potent systemic Th1-type immune responses were induced, but unfortunately the mucosal compartments were not analyzed (209). CTA1-DD/ISCOMs incorporating major outer membrane protein from *Chlamydia muridarum* have also been administered *via* the vaginal route. Vaccination induced limited Ab responses, but clearly detectable CD4 T cell responses in vaginal tissues. This led to protection against a bacterial challenge, as demonstrated by reduction in bacterial shedding from the genital tract (49). Overall, the use of ISCOMs as a delivery vehicle for mucosal vaccination finds its best applicable for nasal administration, even though the sublingual route remains to be explored. Nevertheless, the difficulties related to the use of hydrophilic Ags that have to be modified before incorporation within ISCOMs, together with the reported toxicity of saponin, somehow limits the wide use of such vaccination approach.

### Others

Oil-in-water nanoemulsions disperse into nanosized droplets and exhibit long-term colloidal stability. They can encapsulate hydrophilic or hydrophobic payload, respectively, and have been tested for nasal vaccination. A nanoemulsion based on soybean oil and cetylpiridinium chloride (W805EC) has been shown to deliver its antigenic payload across ciliated nasal epithelial cells and to the regional LNs in the NALT through migrating activated DCs (210). *Via* TLR-2 and TLR-4, such vaccine formulation promoted the induction of robust Ab and Th1-/Th17-type cellular responses and when associated with inactivated Influenza vaccine, generated a protective immunity against Influenza challenge (211). Such an approach similarly proved efficient in animal models to fight against *M. tuberculosis*, Hepatitis B, and *Bacillus anthracis* infections, or to generate HIV-1-specific mucosal immune responses (212, 213). Improved stability of nanoemulsions for mucosal delivery can now be achieved by the double emulsion water-in-oil-in-water technology, which has been applied both nasally and orally and resulted in robust production of systemic IgG and mucosal IgA (214). Alternatively, coupling of lipopeptides with a polylysine core induces the formation of 5–15 nm particles that can promote the generation of systemic and mucosal IgG/IgA and T cell responses after nasal administration. In these conditions, protective responses have been obtained against *S. pyogenes* infection (215, 216).

## Human Application of Lipid Particles for Mucosal Vaccination

Currently approved mucosal vaccines are composed of live-attenuated or killed whole-pathogen cells that offer relatively good efficacy, but cannot be administered to young infants, immunocompromised people and the elderly due to potential safety issues. The majority of mucosally administered vaccines in humans are delivered *via* the oral route and directed against enteric infections such as polio, cholera, typhoid fever, and rotavirus infection (14). Oral polio vaccine has been used for more than 50 years with great success and is a prototypical vaccine for polio eradication in many countries. Interestingly, it demonstrated improved efficacy as compared to an inactivated pathogen vaccine injected parenterally (217–219). Vivotif^®^, as well as Dukoral^®^, Shanchol™, Orochol^®^/Vaxchora™, and mORC-Vax™, are live-attenuated or whole-killed vaccines against *Salmonella*-induced typhoid fever or *Vibrio cholerae* infections, respectively (220–222). As expected based on preclinical studies in animal models, their protective efficacy has been correlated with effector immune responses present at mucosal surfaces (in most cases detection of SIgA in mucosal fluids) and induction of plasma cells expressing gut-homing molecules specific for the small intestine and the colon (223–225). In addition, and similar to observations in animal models, the choice of the administration route impacts on the tropism of the induced mucosal immune responses (226). Only FluMist^®^, a live-attenuated Influenza virus vaccine, is licensed for nasal administration (227). It demonstrates high level of protection against matched and mismatched viral strains in children and adults and proved more efficient than parenteral vaccination (228). Virus-specific mucosal IgA and systemic IgG responses with a possible role for cell-mediated immunity has been documented in vaccinated individuals (229). So far, the only adjuvant used for vaccinal application to mucosae is the B subunit of CT, which has been included in Dukoral^®^ to improve the immunogenicity of the killed whole-pathogen *Vibrio cholerae* vaccine.

Despite many encouraging results obtained in proof-of-concept and preclinical animal models, a limited number of subunit vaccines based on lipidic delivery systems has been evaluated and/or approved for human use, especially for mucosal administration (Table 2) (230). Interestingly, in the context of mucosal vaccination, at least three formulations have been evaluated in early phase clinical trials. Nasal administration of the oil-in-water nanoemulsion W805EC combined with the approved inactivated systemic Influenza vaccine Fluzone^®^ has demonstrated induction of IgA responses in nasal washes in a clinical evaluation (231). In addition, virosome-based and ISCOM-based Influenza vaccines are currently under development for administration *via* the nasal route (232, 233). Systemic injection of virosomes has been licensed for human vaccination against hepatitis A (Epaxal^®^) and Influenza (Inflexal V^®^) (234). Additional vaccines based on such technology have been tested in Phase 1 clinical trial for malaria and Influenza *via* the systemic route (232, 235). The advantage of such approach is that virosomes are self-adjuvanted, which is not the case for all other lipid-based delivery systems. Two Influenza vaccine formulations composed of squalene-based nanoemulsions (MF59^®^ and AS03) are also approved for intramuscular injection in humans, with a particular focus on use in young children and elderly (236, 237). At least three additional strategies have been evaluated in humans. The most promising one is the use of the AS01 adjuvant, composed of liposomes made of highly unsaturated neutral phospholipids including MPLA and saponin QS21 (238). Successful phase III vaccination trials of such delivery system have been performed with the RTS,S/Mosquirix™ vaccine formulation against malaria (239) and the herpes-zooster vaccine (HZ/su) (240). CAF01 is an alternative formulation that is currently tested for parenteral vaccination against tuberculosis. Such liposomal bilayer preparation contains the cationic lipid DDA and the glycolipid TDB as immunostimulator (152). ISCOMs incorporating Ags E6 and E7 from HPV16 have been tested in women with cervical intraepithelial neoplasia and HIV-positive individuals with oncogenic HPV infections (241, 242). They demonstrated a safety profile and induced specific humoral and effector T cell responses. Similar results were obtained following vaccination with HCV-derived antigenic entities (243). Additional vaccine preparations based on lipidic constructs are currently evaluated to fight against pathogen infections, such as malaria, dengue fever, HIV, or Influenza (90, 230).

**Table 2:** Examples of licensed and in development lipid-based vaccines for human use.

	Admin. routes	Clinical situation	Safety, tolerability	Remarks
Liposomes	I.m. I.m. I.m.	Phase 3 trial of AS01 against malaria (239) Phase 3 trial of AS01 against varicella-zooster virus (240) Phase 1 trial of CAF01 against tuberculosis (230)	Safe and well tolerated Safe and well tolerated Unknown to date	Adjuvanted with saponin and monophosphoryl lipid A Evaluated in the elderly Cationic lipid-adjuvanted with trehalose dibehenate
Virosomes	I.m. I.n.	Licensed vaccines against Influenza and Hepatitis (234) Clinical evaluation against Influenza (232)	Safe and well tolerated Unknown to date	Additional formulations in preclinical stages
Immune stimulating complexes	I.m. I.m. I.m. I.n.	Phase 1 trial against HCV (243) Phase 1 trial against HPV (241, 242) Clinical evaluations against Influenza (189) Clinical evaluation against Influenza (233)	Safe, low-mild reactogenicity Safe, low reactogenicity Not reported Unknown to date	Tested in healthy adults and elderly
Microbubbles	I.v.	Not tested in the context of vaccination	Safe and well tolerated	Licensed use for ultrasound-based imaging
Emulsions	I.m. I.m. I.n.	Licensed vaccines against Influenza containing MF59 (236) Licensed vaccines against Influenza containing AS03 (237) Phase 1 clinical trial of W805EC (231)	Some levels of reactogenicity depending on formulations Well-tolerated, no significant adverse events	Additional formulations in clinical evaluation

*I.m., intramuscular; I.n., intranasal; I.v., intravenous*.

Nevertheless, the above-mentioned studies are mainly performed with systemically injected formulations and only rare mucosal applications have been assessed. The use of mucosal route of administration requires that anatomical, functional, and immunological characteristics are taken into consideration and differences between humans and animals may results in poor inter-species translation of promising results (13). An organized NALT similar to that present in mice is not found in humans. By contrast, alternative inductive sites in the form of immune nodules are present in humans in the upper nasal cavity, in the concha, and in Waldeyer’s rings (adenoids, tonsils) (244). Pulmonary delivery of vaccines does not seem to be optimal as well, because BALT have only been reported in fetuses and young children, but not in healthy adults. In addition, the localization and phenotype of DCs in the nasal cavity and in the lung all differ between mice and humans. Taking into account these considerations, sublingual administration sounds like a promising strategy (42). Indeed, immunological and physiological organization of the oral cavity is similar in both mice and humans, with documented presence of the same DC subsets, such as Langerhans cells, capable of eliciting immunogenic or tolerogenic responses depending on the applied formulations. Vaccination *via* the sublingual route has been mostly evaluated in humans for allergen immunotherapy, and has been shown to induce systemic IgG (focus of allergy) (245). Therefore, evaluation of the mucosal vaccination approach to protect against infectious diseases is highly relevant and needs evaluation. In addition, although the gut immune system is relatively similar between both species, oral administration of subunit vaccines is further complicated due to the constraints related to the stability of formulations in the aggressive environment of the digestive tract. On the top of anatomical considerations, the age-related decline in the immune function, possibly related to the documented decrease in M cell differentiation with age, represents a drawback for immunization in eldery people.

## Conclusion and Perspectives

Although vaccination has led to the control of several diseases and has demonstrated substantial technological progresses, humans still suffer from infections leading to death and increasing health costs. Many infectious diseases for which the development of effective vaccines is urgently needed include those transmitted through various mucosal routes that affect the gastrointestinal tract (*E. coli, Salmonella, Shigella, Vibrio cholerae, H. pylori*), the respiratory tract (Influenza, *M. tuberculosis*, respiratory syncitial virus) or are sexually transmitted (HIV, *Chlamydia*) (15). To date, parenteral vaccination represents an important part of the administered vaccines, despite the fact that they poorly induce mucosal immune responses. Furthermore, the requirement for sterile needles, their subsequent elimination, the associated cost, and the cold chain’s requirement in many instances prompt a shift toward reduced frequencies of intramuscular vaccination. In addition, most of the currently licensed vaccines can only be administered over 2 years of age for safety reasons; similar considerations apply for immunodeficient individuals and elderly (13). Therefore, the mucosal application of subunit vaccines represents a sound alternative to broaden the target population that could benefit from vaccination. Cues into this direction include recent advances in the understanding of mucosal immunity as well as assessment of correlates of protection may help to develop promising mucosal vaccines; in this respect, design of novel effective delivery strategies will permit to achieve mucosal vaccines that induce protective neutralizing SIgA, together with CTLs and effector CD4 T cells mainly secreting IFN-γ and IL-17. Moreover, because they are considered as safe, subunit vaccines can certainly be administered to neonates and young infants who already possess a functional mucosal immune system (13).

Lipid-based particles fulfill the requirements for better efficiency, safety, low-dose Ag, and ease to handle logistically. They can deliver a wide range of antigenic entities upon mucosal delivery and can be tailored to obtain vaccine formulations with appropriate properties to address questions related to the mechanisms invlolved in the control of the pathogen and the route of administration. Robust and sustained induction of immune responses, comprising production of SIgA at mucosal surfaces, together with helper and cytotoxic T cells, often correlate with protection in defined animal models under study; this will undoubtly help to drive vaccine development toward the right direction. It remains that a strict comparative analysis of the formulations and administration routes to be used against a particular infectious disease is rarely performed within the same study. To contribute to the identification of such a missing piece in this complex puzzle would be an asset in order to optimize mucosal vaccination. The same lack of information must be filled up when one deals with the definition of the optimal dosing and schedule of administration to ensure efficient priming and boosting of immune responses aiming at reaching optimal magnitude and maintenance.

Some lipid-based preparations have the advantage of being lyophilized, thus allowing to simplify the logistics usually necessary for cold chain. Indeed, some of these formulations can be stored at room temperature for several months and can be administered in such form *via* the nasal, oral, or sublingual routes. Dry powder nasal vaccines have already been tested (246), oral delivery of capsules is not a problem (222), and many allergy-related immunotherapy tablets have been considered for sublingual administration (247). Moreover, lipid-based preparations can be aerosolized, which might represent an alternative procedure to keeping stable vaccine preparations. Overall, great expectations are coming from the lipid-based vaccine formulations currently evaluated in clinical trials in humans, which together with the different mechanisms of mucosal immunity recently unraveled, may likely favor the development of future mucosal vaccines suitable for a majority of individuals, thanks to the combinatorial flexibility offered by the nature of the constituents available.

## Author Contributions

BC and GB planned the manuscript and wrote sections of the manuscript. Both authors read and approved the submitted version of the manuscript.

## Conflict of Interest Statement

The authors declare that the research was conducted in the absence of any commercial or financial relationships that could be construed as a potential conflict of interest.
